# Supplementary material for: The interactome of intact mitochondria by cross-linking mass spectrometry provides evidence for coexisting respiratory supercomplexes
Source: Mol Cell Proteomics. 2017 Dec 8;17(2):216–32. doi: 10.1074/mcp.RA117.000470 (PMC5795388; doi:10.1074/mcp.RA117.000470)
Supplement: Supplemental Data [file supp_RA117.000470_133922_0_supp_23978_fzffwf.zip › spectra_annotation/mito_DR_spectra_annotation/52-1-2-1-10-1.pdf]

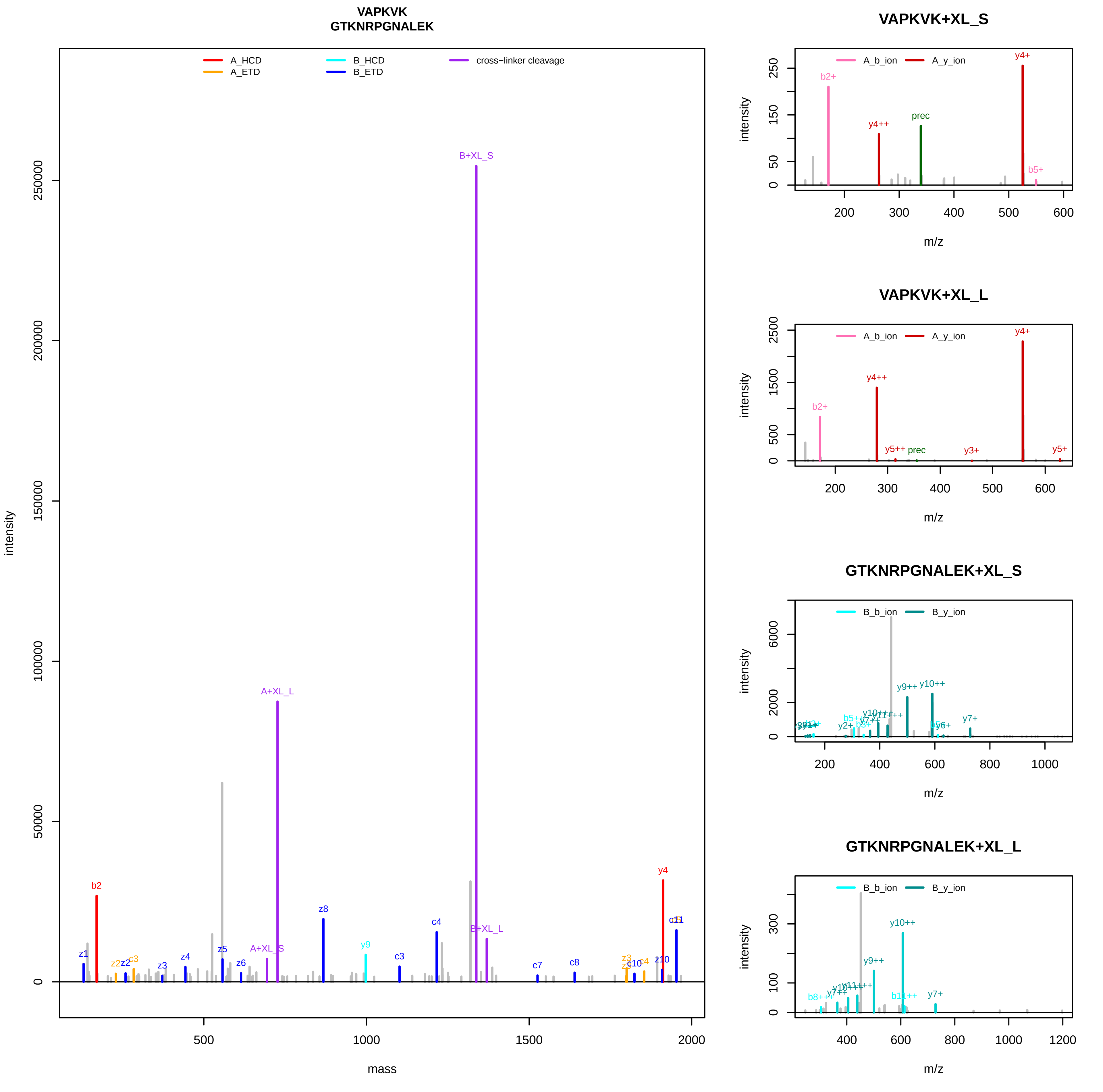

VAPKVK
GTKNRPGNALEK
A_HCD
A_ETD
B_HCD
B_ETD
cross−linker cleavage
intensity
mass
0
50000
100000
150000
200000
250000
500
1000
1500
2000
B+XL_S
A+XL_L
A+XL_S
B+XL_L
b2
y4
y9
z1
z2
z3
z4
z5
z6
z8
z10
c3
c4
c7
c8
c10
c11
VAPKVK+XL_S
A_b_ion
A_y_ion
b2+
y4++
prec
y4+
b5+
m/z
VAPKVK+XL_L
y5++
y3+
y5+
GTKNRPGNALEK+XL_S
B_b_ion
B_y_ion
y9++
y10++
y7+
GTKNRPGNALEK+XL_L
y10++
y9++
y7+
